# Brugada pattern changes during tilt table test with high precordial leads. An exploratory data to better understand the dynamic profile

**DOI:** 10.1016/j.clinsp.2025.100647

**Published:** 2025-04-04

**Authors:** Eduardo Nolla Silva Pereira, Luciana Sacilotto, Tan Chen Wu, Gabriele D'Arezzo Pessente, Denise Tessariol Hachul, Mauricio Ibrahim Scanavacca, Francisco Carlos da Costa Darrieux

**Affiliations:** Instituto do Coração ‒ (InCor), Faculdade de Medicina, Hospital das Clínicas da Universidade de São Paulo (HCFMUSP), São Paulo, SP, Brazil

**Keywords:** Brugada syndrome, ST-segment elevation, Autonomic nervous system, Tilt table test

## Abstract

**Introduction:**

Brugada Syndrome (BrS) typically manifests with a dynamic type 1 Brugada pattern (Br1ECGp) that depends on the dominance of the sympathetic or parasympathetic nervous system. This pattern is more prominent during activities that stimulate the vagus nerve, such as sleeping and after meals. By conducting the tilt table test and adding high precordial leads to the standard protocol, it is possible to provoke sympathetic or parasympathetic responses, providing valuable information.

**Materials and methods:**

This was an observational, cross-sectional and hypothesis-generating pilot study. The tilt table test was performed with high precordial leads on three Brugada patients to assess the Br1ECGp during the test. The ECG features were continuously recorded to analyze variations during all phases of the test.

**Results:**

The first patient exhibited a significant Br1ECGp that disappeared during the upward phase of the tilt table test and reappeared during the recovery phase. The second patient had a Br1ECGp in three leads that persisted during both the upward and recovery phases of the tilt table test. The third patient displayed a Br1ECGp in three leads, which was maintained during the upward phase in two leads and recovered during the recovery phase. The third patient was the only one to experience cardioinhibitory syncope.

**Conclusion:**

This study provided insights into the dynamics of the Br1ECGp during the tilt table test when enhanced by high precordial leads. It reaffirmed that the Brugada ECG pattern is influenced by sympathetic or parasympathetic dominance.

## Highlights

• Brugada syndrome presents with a dynamic ECG pattern during autonomic stimulation.
• Tilt table test can provide different autonomic situations during the exam.
• High precordial leads can be used during the test to enhance ECG information.

## Introduction

Brugada Syndrome (BrS) is an autosomic dominant inherited disease more frequently affecting male adults.^1^ It is characterized by the presence of type 1 Brugada ECG pattern (Br1ECGp), which is defined as a coved-shaped 2 mm ST-segment elevation in at least V1 or V2 on a 12-lead Electrocardiogram (ECG), in standard or High Precordial Leads (HPL).^2^

Ventricular fibrillation and Sudden Death (SD) occur mainly during sleep and rest.^3^ The typical ECG patterns are dynamic and modulated by exercise or pharmacologic interventions that interact with the autonomic nervous system or the cardiac sodium channel (e.g. ajmaline, flecainide, and procainamide).^3^, ^4^, ^5^

The pursuit of the Br1ECGp is significant due to its prognostic impact.^6^ However, it is complicated by the variability in its pattern.^3^, ^4^, ^5^ High Precordial Leads (HPL) have been used to elicit the Brugada pattern for increasing sensitivity when screening.^7^ The authors hypothesize that performing the tilt table test, which also tests the vagal component, may provide a window of opportunity for potential unmasking or augmentation of this BrS pattern.

The authors performed tilt table test on 3 patients with BrS using high precordial leads to evaluate the dynamic changes during the test. All patients signed a consent form, and this pilot protocol was approved by the local ethics committee.

## Material and methods

This study was performed in accordance with the principles of the STROBE Statement. The patients signed an informed consent form, and the study protocol was approved by the local ethics committee (CAE 5422,341.8.0000.0068, CAPPesq – HCFMUSP, on 05/01/2022).

The patients were randomly selected from the ambulatory clinic of a tertiary hospital in São Paulo, Brazil. The inclusion criteria were the presence of the Br1ECGp pattern during the initiation of the test, being over 18 years old, and signing the informed consent.

Due to the rarity of the disease, only three patients could be included in this pilot study. The tilt table test was performed on the three BrS patients using high precordial leads to assess the dynamic changes during the test.

**Fig. 1:**
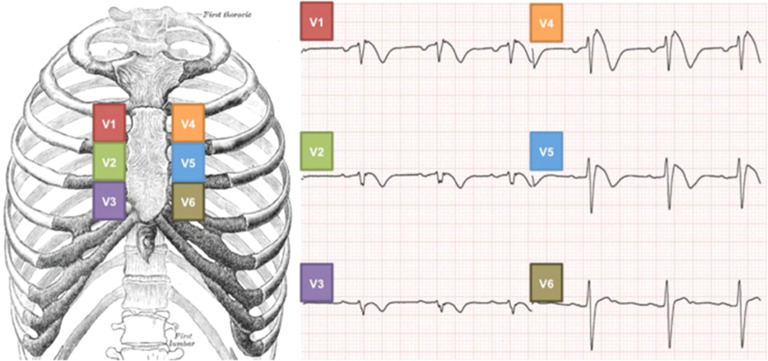
High precordial leads using the respective second, third e fourth intercostal spaces. He presented a Br1ECGp in V2^nd^R, V3^rd^R, V4^th^R, V2^nd^L and V3^nd^L (black arrow). During table upward phase, all Br1ECGp disappeared, however, the Br1ECGp returned in the same derivations (blue arrow). Br1ECGp, type 1 Brugada ECG pattern.

The tilt table test was conducted according to the local protocol, with some adaptations, as follows. Blood pressure was measured every 2 min and with continuous oximetry. The ECG was performed with high precordial leads (Fig. 1). The V1 lead is placed at V2^nd^R, V2 is placed at V3^rd^R, V3 is placed at V4^th^R, V4 is placed at V2^nd^L, V5 is placed at V3^rd^L, and V6 is placed at V4^th^L. The patient remains in the supine position for 5 mins. The table is tilted up to 60°‒80° When nitrate is used, the passive tilt test lasts 20 mins, then the authors administer 1.25 mg of sublingual dinitrate isosorbide, and the position is maintained for another 20 mins. If nitrate is not used, the tilt test lasts 30 mins. If symptomatic hypotension/bradycardia happens, the authors promptly return the table to the supine position.

Fig. 1 High precordial leads using the respective second, third and fourth intercostal spaces. (with permission).^8^

## Results

The first patient was a 57-year-old male with Brugada syndrome. He was asymptomatic and had lost multiple family members to SD, including his father, uncle, cousins, and nephew during sleep. The genetic test found no pathogenic mutations. He discovered his disease during a consultation in the emergency department for other reasons.

**Fig. 2:**
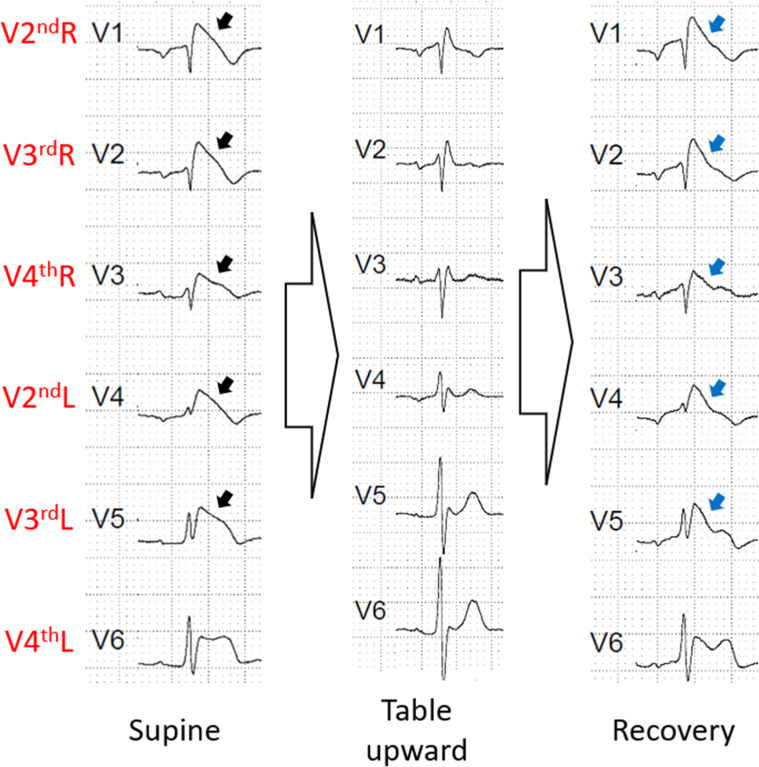
The ECG phases of the first patient's exam. He presented a Br1ECGp in V2^nd^R, V3^rd^R, V4^th^R, V2^nd^L and V3^rd^L (black arrow). During table upward phase, all Br1ECGp disappear, however, the Br1ECGp returned in the same derivations (blue arrow). Br1ECGp, type 1 Brugada ECG pattern.

The tilt test was done without nitrate. During the supine phase, the patient had Br1ECGp from V2^nd^R to V3^rd^L. In the table upward phase, he had no Br1ECGp. No nitrate was administered. He had no symptoms of hypotension/bradycardia during the exam. During the recovery phase, the pattern returned very similar to the first phase with the Br1ECGp from V2^nd^R to V3^rd^L (Fig. 2).

The second patient was a 32-year-old male with Brugada syndrome. He had an Implantable Cardioverter Defibrillator (ICD) due to an arrhythmic syncope he suffered in 2006. His cousin had a sudden death at the age of 23. He had inappropriate shocks, but never an appropriate one. He carried a pathogenic mutation at the SNC5A gene (p.Y1009*).

**Fig. 3:**
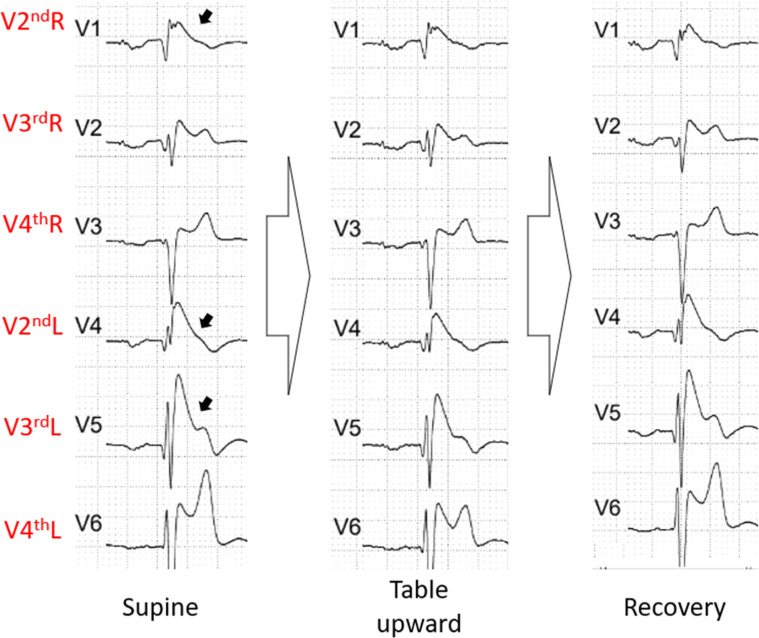
The ECG phases of the second patient's exam. He presented a Br1ECGp in V2^nd^R, V2^nd^L and V3^rd^L (black arrow). No alteration was seen during the exam. Br1ECGp, type 1 Brugada ECG pattern.

The test was perfrormed without nitrate. During the supine phase, the patient exhibited Br1ECGp in V2^nd^R, V2^nd^L and V3^rd^L, and a first-degree atrioventricular block as well. During the whole exam, he maintained the same pattern (Fig. 3).

The third patient was a 59-year-old male with Brugada syndrome. He also had an ICD due to an arrhythmic syncope and had received appropriate shock therapy. There was no SD in his family, and the genetic testing found no pathogenic mutation.

**Fig. 4:**
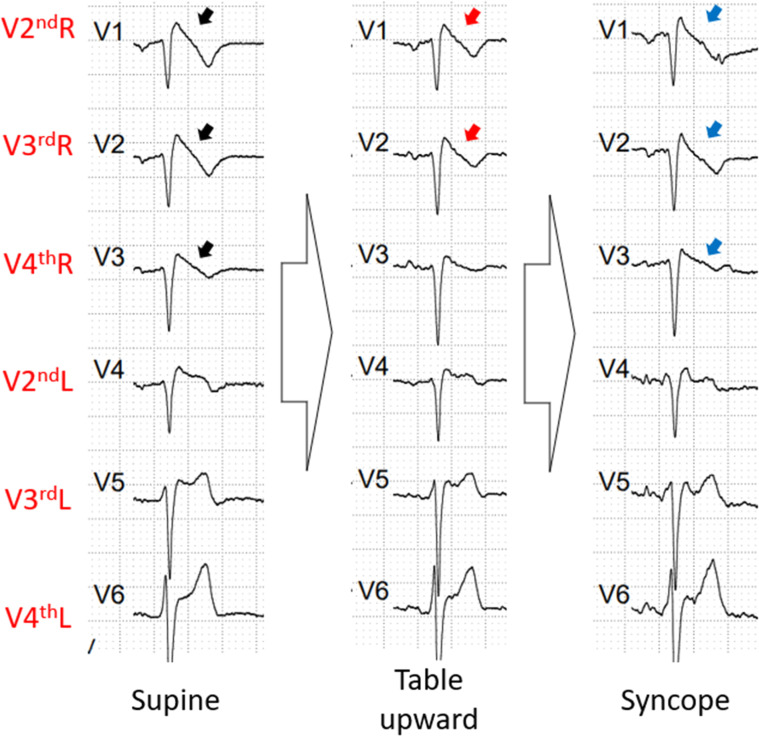
The ECG phases of the third patient's exam. He presented a Br1ECGp in V2^nd^R, V3^rd^R, V4^th^R (black arrow). During table upward phase, V2^nd^R and V3^rd^R maintained the Br1ECGp, however V4^th^R (red arrow) had a reduced ST elevation to a <2 mm. After the syncope, the Br1ECGp returned from V2^nd^R to V4^th^R (blue arrow). Br1ECGp, type 1 Brugada ECG pattern.

The test was performed with nitrate. During the supine phase, the patient exhibited Br1ECGp from V2^nd^R to V4^th^R. However, In the table upward phase, the Br1ECGp disappeared in lead V4^th^R due to ST elevation no longer than 2 mm. He had a cardioinhibitory syncope, and when he was laid down, the Br1ECGp returned from V2^nd^R to V4^th^R (Fig. 4).

## Discussion

To the best of our knowledge, this is the first study involving patients with BrS submitted to a modified tilt table testing with HPL. In these three first cases, the exam was not able to augment the Brugada pattern, however it showed the dynamic pattern of BrS. The most dynamic Brugada pattern was observed in the first case, reaching no Br1ECGp in the orthostatic position. One might argue that this could be the “worst” Br1ECGp, however, it is extremely dynamic, and the patient was asymptomatic. In the second case, only in the HPL was the Br1ECGp seen, and a minimal variation of this pattern was observed. In the third case, the variation was very subtle, however, the Br1ECGp disappeared in V4^th^R due to an ST-elevation less than 2 mm. The two latter cases were symptomatic, suggesting that maybe the absence of dynamicity can be worse than the pattern itself. These issues still require futher case studies to be validated.

A possible cause for the ST variation could be due to the positional change. Indeed, Markendorf et al.^9^ evaluated heart rate, QT interval, QTc interval, T-wave vector and direction, and ST-segment deviation in supine and upright positions from 1,028 patients. Positional change from supine to upright resulted in an increased heart rate, reduced QT interval, increased QTc interval, variation in T-wave direction, and ST-segment elevation in 0.4 % and ST-segment depression in 0.2 %. Such a small ST-segment deviation in this study, would make it improbable that the changes seen in the present study would be due only to positional change. Another point is that the variations presented in this study did not occur immediately after the positional change, they appeared after a few minutes in the table's upward position.

The Br1ECGp disappearance during table upward was physiopathology expected. During the upward position in the tilt table test, there is a reduction in the ventricular filling causing an immediate fall in arterial pressure. The carotid sinus and aortic arch baroreceptor induce an increase in the heart rate and sympathetic tone.^10^ This can explain the Br1ECGp disappearance.

Mizumaki et al.^3^ reported that augmentation of ST elevation (≥ 1.5 mm/20 min) occurred more frequently during 24-hour monitoring in symptomatic Brugada patients. The maximum ST elevation was accompanied by an increase in high-frequency power (HF 0.15‒0.4 Hz) and the RR interval, along with a decrease in the ratio of the low-frequency component (LF 0.04‒0.15 Hz) to the High-Frequency component (LF/HF).

The ECG signs of BrS can be augmented by the selective stimulation of alpha-adrenoceptors, muscarinic receptors, or IA antiarrhythmic class drugs, but they can be reduced using drugs such as beta-adrenoceptors stimulation or alpha-adrenoceptor blockade.^11^ Indeed, isoproterenol has been used to treat ventricular arrhythmias and electrical storms associated with multiple ICD shocks in Brugada patients.^12^^,^^13^

Bigi et al.^14^ investigated the role of Cardiac Autonomic Neuropathy (CAN) risk stratification in 28 patients with Br1ECGp and 87 non-type 1 BrS. Four standard cardiac autonomic tests were performed and the presence of at least 2 were considered positive for CAN. None of the non-type 1 patterns had CAN. Among the patients with Br1ECGp, CAN was detected in 13 (46%). Of 13 patients with CAN, 11 (84%) had previous cardiac events compared with only two of 15 patients (13%) without CAN. This study found no difference in risk stratification based on sex category.

Other situations involving the autonomic system have been tested. Recently, a new treadmill exercise protocol to unmask the Br1ECGp during the recovery phase and using HPL was tested. Pichara et al.^5^ reported that this test increased by 32.4% the diagnostic yield compared to HPL alone. Another test evaluated is the “full stomach test”, using large meals as a stimulation. These studies enhanced diagnostic accuracy in patients with Brugada syndrome.^15^^,^^16^

The first observations may generate new hypotheses regarding the complexity of mechanisms and risk profiles in BrS. The authors will need to extend the initial observations in patients with a suspicious BrS profile and perhaps incorporate family members screened for this purpose into the routine.

## Conclusions

These findings strongly highlight the role of the autonomic nervous system in the pathophysiology and potencial risk stratification of Brugada syndrome. Parasympathetic tone can influence ECG patterns and may play a significant role in the occurrence of arrhythmias. These autonomic-focused tests may have the potential to become incremental diagnostic tools, as they provide unmasking of the Br1ECGp, as well as identification of patients who could benefit from an ICD.

## Authors' contributions

All authors read and approved the final manuscript.

E. Nolla S. Pereira: Conceptualization; Methodology; Investigation; Writing-Original draft preparation.

L. Sacilotto: Data curation; Software; Reviewing and Validation.

T. Chen Wu: Visualization; Investigation.

G. D. Pessente: Investigation; Visualization.

D. T. Hachul: Visualization; Validation.

M. I. Scanavacca: Supervision; Validation.

F. C. C. Darrieux: Supervision; Project Management; Reviewing and Editing.

## Funding

This research did not receive any specific grant from funding agencies in the public, commercial, or not-for-profit sectors.

## Financial disclosure

The authors do not have any financial agreement with a company whose product is prominently featured in the present manuscript or with a company that produces a competing product.

## Declaration of competing interest

The authors declare no conflicts of interest.
